# Agronomic drivers of Tribenuron-methyl resistance in wild mustard (*Sinapis arvensis*) in dryland wheat fields of western Iran

**DOI:** 10.1371/journal.pone.0358794

**Published:** 2026-09-24

**Authors:** Fattah Moradi, Alireza Bagheri, Farzad Mondani, Iraj Nosratti

**Affiliations:** Department of Plant Production and Genetics, Razi University, Kermanshah, Iran; Institut National de la Recherche Agronomique, MOROCCO

## Abstract

Understanding the impact of common agronomic practices on the evolution of herbicide resistance is essential for developing sustainable weed management strategies that incorporate non-chemical control methods. In this study, we investigated the frequency of Tribenuron-methyl (TBM) reduced-sensitivity in wild mustard (*Sinapis arvensis* L.) by analyzing survival patterns and their association with agronomic management across 313 dryland wheat fields in the Eslamabad-e-Gharb district, Kermanshah, Iran. The results showed that survival levels in the studied fields varied notably, with 47.3% exhibiting survival between 0% and 20%, 14.0% falling within the 20% to 30% range, and 38.7% showing survival above 30%, highlighting the diverse survival patterns across the region. Logistic regression analysis identified intensive TBM application as the strongest factor promoting survival proportion, with triennial applications (3×) over a five-year period increasing the risk of reduced-sensitivity in wild mustard more than tenfold (Exp(B)=10.651) compared to no use. The selection of wheat cultivar also played a significant role; using Azar-2 raised the survival by more than twofold (Exp(B)=2.218) relative to Sardari. Furthermore, field area significantly influenced survival dynamics, with larger farms (15 ha and more) showing higher mean survival proportions (42.1%) and greater frequencies of intensive TBM application. Conversely, incorporating chickpea into the crop rotation significantly reduced survival proportions. These findings highlight the necessity of adopting diversified agronomic practices—including herbicide rotation, crop rotation, and competitive cultivar selection—to mitigate herbicide resistance frequency. Furthermore, strengthening grower awareness regarding resistance mechanisms and improving the outreach of effective management protocols are critical.

## 1. Introduction

The widespread and repeated use of herbicides with the same mode of action has led to significant selective pressure on weed populations, resulting in the evolution of herbicide resistance in many species [1]. This phenomenon has become a major challenge for agricultural producers, complicating weed management and threatening crop yields [2]. Beyond agronomic concerns, herbicide resistance has important ecological implications, including shifts in plant community composition and the potential for resistance gene flow to related species, which can disrupt local ecosystems (Schütte et al., 2017). Increased herbicide use to manage resistant weeds also raises the risk of environmental contamination, contributing to soil degradation, water pollution, and loss of biodiversity [3–5]. Economically, herbicide resistance increases production costs due to yield losses, weed seed contamination, reduced land value, and the need for alternative management strategies [6].

Globally, herbicide resistance has been documented in 273 weed species across 75 countries and 102 crops, with wheat cultivation accounting for a substantial proportion of reported cases [7]. In Iran, where wheat is a staple crop and chemical weed control is the dominant management strategy, the emergence of herbicide-resistant weeds poses a significant threat to food security and farmer livelihoods [8]. Recent global surveys indicate that resistance has been confirmed in multiple weed biotypes across several provinces, including Kermanshah, predominantly affecting cereal production systems. Notably, five biotypes have shown resistance to acetolactate synthase (ALS) inhibitors, a widely used herbicide group in cereal production [7].

ALS inhibitor herbicides, such as TBM, have been favored for their high efficacy at low doses and relatively low mammalian toxicity [9]. However, their extensive use has resulted in strong selection pressure and the rapid evolution of resistance, making ALS inhibitors the herbicide group with the highest number of resistant weed species globally [7]. TBM resistance has been reported in 49 weed species worldwide, including wild mustard (*Sinapis arvensis* L. [syn. *Mutarda arvensis* (L.) D.A. German]), which is a major weed in wheat fields in Iran and several other countries [7].

Multiple studies have documented TBM-resistant wild mustard populations in Iran, with over 50% of individual plants in certain populations surviving herbicide application, thereby indicating high resistance levels in these populations [10,11]. These findings underscore the urgency of addressing herbicide resistance in Iranian wheat production systems. While much of the existing research has focused on identifying resistant biotypes and elucidating resistance mechanisms, less attention has been given to the role of agronomic practices in driving resistance evolution at the field or landscape scale. Although continuous wheat cultivation and a lack of herbicide rotation frequently correlate with high resistance levels [12,13], systematic investigations linking specific management practices—such as crop rotation, herbicide rotation, tank mixtures, and certified seed use—to resistance evolution remain scarce.

Integrated weed management strategies, including crop and herbicide rotation, are recognized effective approaches for delaying or mitigating herbicide resistance [14–17]. In addition, crop competitiveness plays an important role in resistance management, as cultivars with traits such as early vigor and high tillering can suppress weed growth and reduce herbicide dependence [18,19]. This approach decreases weed seed production and soil seed banks, enhancing the sustainability of integrated weed management programs. Nevertheless, the adoption of such practices remains limited, and many farmers continue to rely primarily on chemical control. Understanding how farming practices contribute to resistance evolution is essential for informing management recommendations and encouraging the adoption of sustainable alternatives.

This study addresses these gaps by evaluating the association between common agronomic practices and the frequency of TBM-resistant wild mustard populations in dryland wheat fields across western Iran. By integrating multi-year management records with field surveys of resistance, we aim to identify the key drivers of resistance evolution and highlighting practical interventions that can reduce selection pressure. The findings will inform both farmers and policymakers about effective strategies for managing herbicide resistance, supporting the transition toward more sustainable weed management in Iranian wheat production.

## 2. Materials and methods

**Fig 1:**
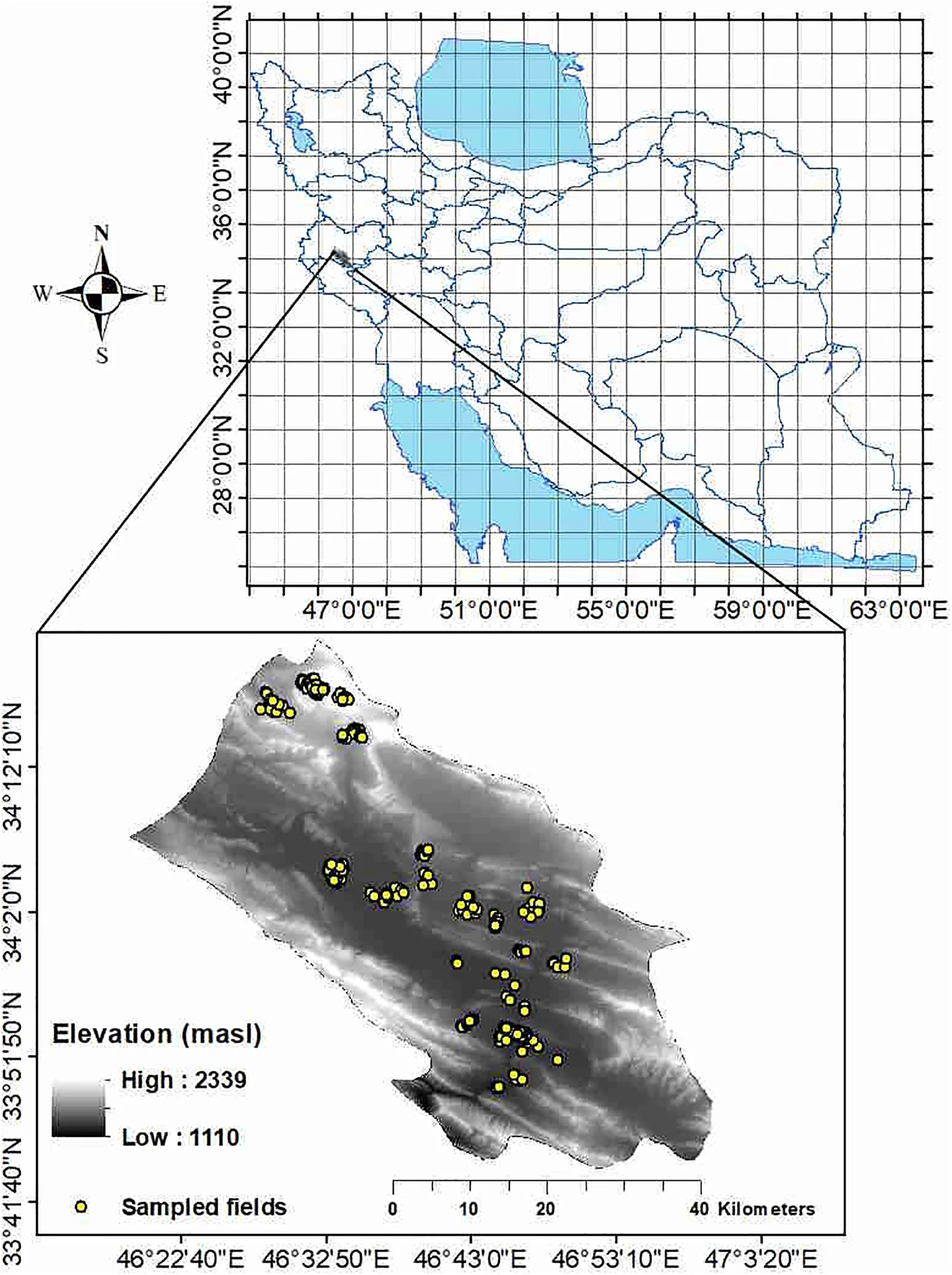
Geographic location of the study area within Iran and spatial distribution of sampled dryland wheat fields (yiellow dots). This map is entirely original, created directly by the authors using our own field survey data and standard open-source vector boundaries and does not contain copyrighted.

The study was conducted in dryland wheat fields across the Eslamabad e Gharb districts in Kermanshah, Iran (Fig 1), which is characterized by a temperate Mediterranean climate with an average annual rainfall of 453 mm. According to the 2022 agricultural census, 61.9% of the agricultural land in this area is dedicated to wheat cultivation. Long-term meteorological data used for this study were obtained from the Iran Meteorological Organization.

The study comprised two interconnected components: a sampling survey followed by a controlled greenhouse experiment. The sampling survey was conducted initially to collect seed samples of wild mustard from various farms and to gather essential data on agronomic practices. This included information on agronomic management history over the past five years, such as crop rotation, tillage methods, seed sources, fertilizer types, wheat cultivar, seeding rate, sowing date, sowing method, and weed management strategies. Additionally, social factors such as farmers’ experience, literacy level, and farm size were documented (see Table 1).

**Table 1:** Agronomic management and farmer demographics surveyed.

Agronomic factors	Responses
Number of chickpea cultivation over the last 5 years	Two times/ three times
Existence of fallow in the crop rotation	Yes/ No
Number of crops in rotation	Two/ three/ four
Tilling before sowing	Yes/ No
Type of tillage	No-tillage/ moldboard/ harrow/ moldboard and harrow
Type of fertilizer	Nitrogen and phosphorus/ phosphorus/ potassium and phosphorus
Seed source	Saved seed/ Purchased seed
Wheat cultivar	Sardari/ Azar-2
Seeding rate	140 to 150/ 151–160/ 161–170 (kg ha^-1^)
Sowing date	Early October/ early November
Sowing method	Hand spreading/ Row crop planter
Herbicide application date	February/ March
Tribenuron-methyl application frequency over the last 5 years	No use/ one time/ two times/ three times
Combination of Tribenuron-methyl with 2,4-D	Yes/ No
**Farmers demographics**	**Responses**
Farmer experience (years)	10 to 20/ 21–30/ 31–40/ 41–50/ 50 and above
Farmer education level	Illiterate/ elementary/ middle/ high school/ bachelor and above
Farmer occupation	Only farming/ farming plus a second job
Field area	1 to 4.9/ 5 to 9.9/ 10 to 14.9/ 15 and more (ha)

Comprehensive data collection was achieved through face-to-face interviews with farmers, supplemented by follow-up interviews to enhance data reliability. To ensure questionnaire validation and data reliability, the structured questionnaire was initially reviewed and refined by a panel of agronomy and weed science experts and pre-tested through a pilot survey with 15 local farmers. Furthermore, to minimize recall bias regarding the five-year retrospective data, we highlight that crop rotation in the drylands of the studied region is relatively simple, predominantly comprising a wheat–chickpea rotation, with barley and lentils occurring only occasionally. The agricultural management program for each crop is routine and repeated annually. In addition, farming operations are largely dictated by the region’s available machinery and implements, combined with deeply ingrained management habits developed over years of practice. Consequently, gathering five-year historical information from farmers was structured around the specific crop types, their sequential order in the rotation, and these established operational routines. Because farmers follow consistent and repetitive management practices for each crop, the structural simplicity of these dryland systems inherently facilitates accurate historical tracking.

### 2.1 Collection of plant material

A total of 313 farms were randomly selected and surveyed using a random number generator [20] (Fig 1). Sampling was carried out on the selected farms by identifying 15 distinct wild mustard patches using a zigzag sampling pattern during the seed ripening stage. From each patch, the terminal parts of up to 10 plants, specifically the siliques, were collected and stored in paper bags. The samples were transported to the Plant Physiology Laboratory of Razi University where seeds were manually extracted from the siliques. Seed samples from all patches within each farm were then combined to form a composite seed batch, representing the seed population of that farm, and used for subsequent survival-based resistance screening experiment to evaluate reduced sensitivity and field resistance.

Prior to seed collection, preliminary plant samples of wild mustard were transferred to the Herbarium of Agricultural and Natural Resources Campus, Razi University, and authenticated by a resident herbarium specialist. Based on these verified reference and standard botanical keys, field identification was accurately carried out using key morphological characteristics, upper leaf morphology, inflorescence structure, and particularly silique shape and surface texture, and seed morphology.

Field sampling was conducted with the verbal informed consent of the private landowners. No formal governmental or legal permits were required for the collection of *Sinapis arvensis* plant material, as the species is an agricultural weed targeted for management and control, and it is not a protected, endangered, or legally restricted plant. Furthermore, since this study involved weed surveys and sampling of agricultural weeds on private farmlands without involving human participants or clinical trials, formal institutional review board approval was not required.

### 2.2 Discriminatory dose determination

A preliminary dose-response bioassay was performed using a reference susceptible wild mustard population to establish an appropriate discriminatory dose for resistance screening. Tribenuron-methyl 75% DF was applied at eight concentration levels: 0 (non-treated control), 3.75, 7.5, 15, 30, 60, 120, and 240 g ai ha^−1^ [21]. Herbicide applications were made at the 2–4 true leaf stage, with plant dry biomass evaluated 28 days post-treatment. Dose-response data were analyzed using a three-parameter logistic regression as described by Ritz and Streibig [22]. The resulting model parameters (upper limit = 51.40, GR₅₀ = 8.82 g a.i. ha^−1^, slope = 4.06) were used to calculate the effective dose providing a 90% growth reduction (GR₉₀ = 14.60 g ai ha^−1^). Based on these results, a discriminatory dose of 20 g ha^−1^ TBM was selected for population screening.

### 2.3 Seed preparation and bioassay setup

Seeds collected from 313 wild mustard field populations underwent standardized preparation protocols. Dormancy was broken through cold stratification at 5°C for 14 days, following optimization trials conducted in preliminary experiments in accordance with Sharifi and Goldani [23]. Seeds were surface-sterilized by immersion in a 1% sodium hypochlorite solution for 2 minutes, then rinsed thoroughly with sterile distilled water [24]. Fifteen seeds per population were sown in 12-cm-diameter black plastic pots containing clay-silty loam soil sourced from an herbicide-untreated location. After seedling emergence, the plants were thinned to 10 uniform seedlings per pot, and the remaining ones were discarded.

### 2.4 Resistance screening

The survival rate and reduced sensitivity status of 313 wild mustard populations were determined using a survival-based, single-dose bioassay, following the methodology of Moss et al. [25], Sin and Kadıoglu [26]. The experiment was conducted in two independent runs under controlled greenhouse conditions (25/13°C day/night, 12-hour photoperiod) [27]. Within each run, pot positions were completely randomized using a computer-generated number sequence to minimize positional effects. A linear mixed-effects model was used to account for potential variability between farms and runs. “Farm” was treated as a fixed effect to evaluate differences in resistance percentages among fields, while “Run” was included as a random effect to account for temporal replication.

At the 2–4 true leaf stage, plants were treated with a discriminatory dose of TBM at 20 g ha^−1^. The application was performed using a calibrated HYUNDAI HP 1690 sprayer fitted with a flat-fan nozzle, delivering a spray volume of 400 L ha^−1^. To ensure uniform application and reproducibility, the spray boom was maintained at a constant height of 50 cm above the plant canopy using a physical guide rod, and the application speed was strictly standardized and controlled at 1 m s^−1^ across all plots. Plant survival was assessed 28 days after treatment by counting the number of surviving individuals in each pot. The survival rate for each population was calculated, and the final survival proportion was based on the mean value from the two experimental runs. To classify populations as resistant or susceptible, we evaluated the level of injury based on a scale of 0–100, where 0 represents complete plant death and 100 represents no injury. Following Davis et al. [28], populations with a survival proportion > 70% were categorized as resistant, while those ≤ 70% were classified as susceptible.

### 2.5 Data analysis

Considering the use of unstructured qualitative questionnaires in this experiment, data analysis focused on the effect of each agronomic practice and farmer characteristics as independent variables on the wild mustard survival against TBM as the dependent variable [29]. Since the recorded survival was a proportion and did not follow a normal distribution, non-parametric tests were employed to analyze the data. Thus, the Mann-Whitney U test was used to compare differences between two groups of independent variables (Number of chickpea cultivation during the last five years in rotation, existence of fallow in the last five-year crop rotation, tillage before sowing, seed source, wheat cultivar, sowing date, sowing method, weed management, herbicide combination, as well as farmer occupation situation— see Table 1). The Kruskal-Wallis H test was applied to measure the effect of multi-group independent variables (number of crops in the five-year rotation, type of tillage, fertilizer type, sowing rate, herbicide type, application of TBM during the last five years, as well as farm size, farmer experience, and education levels— see Table 1) on the survival proportion of wild mustard to TBM. Subsequently, to assess the combined effects of the significant variables identified in the non-parametric preliminary screening, a binary logistic regression model was employed. For this regression, the resistance status was treated as a binary response variable (present/absent), using the 70% classification threshold. Prior to fitting the model, a multicollinearity check was performed by calculating Variance Inflation Factors (VIF) and tolerance values for the input independent variables, confirming the absence of multicollinearity. This combined approach of univariate screening allowed us to evaluate the contribution of multiple management practices simultaneously. In order to evaluate the relationship between effective agronomic activities with farmer characteristics, Chi-square tests of independence were applied, and the significant relationships were discussed [29].

## 3. Results

### 3.1 Effect of agronomic practices on field resistance

The results of linear mixed-effects model revealed that the effect of farm was highly significant (F = 17.790, p < 0.001), indicating substantial variability in survival rates across farms. This finding supports the primary objective of the study, which was to identify reduced-sensitivity populations across the fields. The effect of run yielded a negligible variance estimate (0.33, SE = 0.85) compared to the residual variance (85.61), indicating that survival proportions were consistent across runs, justifying the combination of data from both runs for final survival rate estimates.

**Fig 2:**
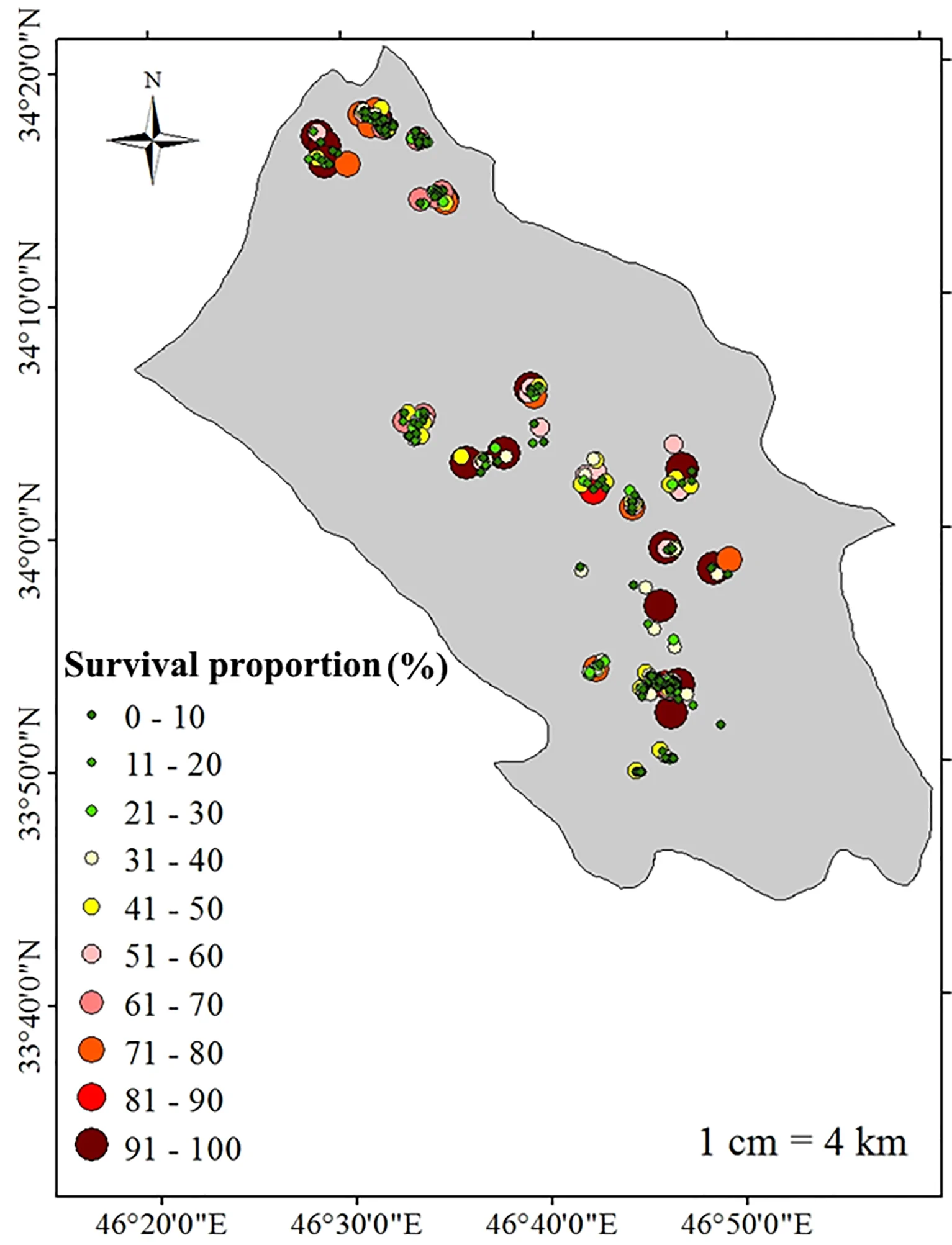
Spatial frequency and distribution of survival proportions of wild mustard (*Sinapis arvensis*) populations in sampled dryland wheat fields following tribenuron-methyl (TBM) application. Survival categories are based on seedling survival percentages under screening dose assays.

The distribution of TBM reduced-sensitivity in wild mustard populations varied significantly across the sampled dryland wheat fields (Fig 2). Nearly half of the surveyed fields (47.3%) exhibited survival levels of 0–20%, with the vast majority of this group (39.6%) showing minimal survival (0–9%). Approximately 14.1% of fields demonstrated survival levels of 20–29%. Notably, a substantial portion of the fields (38.7%) presented survival rates exceeding 30% of plants surviving the herbicide treatment. Other survival categories were further distributed as follows: 9.6% of fields showed 30–39% survival, 9.6% exhibited 40–49%, 6.4% had 50–59%, 4.5% demonstrated 60–69%, 3.5% showed 70–79%, and 5.0% of fields displayed extremely high survival (80–100%), including 3.8% with complete (100%) survival (Fig 2).

Agronomic factors exhibited pronounced effects on survival dynamics. The number of chickpeas cultivation during the last five years in rotation, fertilizer type, wheat cultivar, sowing date, and application of TBM during the last five years had significant effects on the survival proportion of wild mustard to TBM (Table 2).

**Table 2:** Results of the Mann-Whitney U Test and Kruskal-Wallis H test for comparing two and multiple independent groups of agronomic practices.

Non-Parametric Tests	Groups	df	Test statistics	P-value
Mann-Whitney U test	Number of chickpea cultivation in the last 5 years	1	10031.50	0.045
	Existence of fallow in the crop rotation	1	2978.00	0.574
	Tilling before sowing	1	11977.50	0.881
	Seed source	1	9578.00	0.516
	Wheat cultivar	1	7290.50	0.025
	Sowing date	1	9391.50	0.005
	Sowing method	1	11044.00	0.130
	Herbicide application date	1	11119.50	0.483
	Combination of Tribenuron-methyl with 2,4-D	1	9730.50	0.204
Kruskal-Wallis H test	Number of crops in rotation	2	0.928	0.629
	Type of tillage	3	1.374	0.712
	Type of fertilizer used	2	7.671	0.022
	Seeding rate	2	4.888	0.087
	TBM* application frequency over the last 5 years	3	15.220	0.002

* TBM= Tribenuron-methyl.

Descriptive statistics provided granular insights into survival and reduced sensitivity trends across agronomic factors. There was a significant increase in the frequency of surviving populations of wild mustard with increasing frequency of TBM application (Table 3). Wheat cultivar selection emerged as a pivotal factor, with Azar-2 plots showing a higher mean survival proportion than Sardari. Increasing the frequency of chickpea cultivation in the crop rotation over the last five years reduced the survival proportion of wild mustard to TBM. The proportion of wild mustard survival following TBM application was lower in farms with a history of three chickpea crops in rotation compared to those with two. Sowing date further modulated survival dynamics, with November-sown fields recording survival rates 8.2% higher than October-sown fields. Furthermore, survival proportions were lower in fields receiving phosphorus and potassium fertilizers combined, compared to those treated with nitrogen-phosphorus or phosphorus-only fertilizers (Table 3).

**Table 3:** Descriptive statistics comparing median, ranks, and mean of wild mustard resistance to Tribenuron-methyl (TBM) across different groups of agronomic practices.

Variable	Agronomic factors	Median (%)	Mean Rank	Resistance proportion ± SE*
Number of chickpea cultivation in the last 5 years	Two times	25.00	169.70	30.5 ± 2.7
	Three times	20.00	149.21	24.1 ± 1.9
Wheat cultivar	Sardari	20.00	150.88	24.7 ± 1.8
	Azar-2	32.22	177.13	32.58 ± 3.4
Sowing date	October	16.67	146.16	23.4 ± 1.9
	November	29.29	174.91	31.6 ± 2.6
Type of fertilizer used	K&P	0.00	106.38	12.4 ± 5.1
	P	22.22	157.01	25.5 ± 2.8
	N&P	25.00	162.03	28.3 ± 2.0
TBM application frequency over the last 5 years	No use	15.83	144.42	24.1 ± 7.1
	One time	20.00	150.39	24.3 ± 1.9
	Two times	22.22	158.75	26.7 ± 3.1
	Three times	42.86	231.53	51.4 ± 6.6

* SE= Standard Error.

The binary logistic regression model was employed to evaluate the simultaneous effects of key agronomic practices on the survival proportion (Table 4). Prior to model fitting, collinearity diagnostics confirmed the complete absence of multicollinearity, as all VIF values ranged between 1.012 and 1.106, with high Tolerance values exceeding 0.90 for all predictors. Furthermore, the overall goodness-of-fit and explanatory power of the model were highly satisfactory, as demonstrated by the Omnibus Tests of Model Coefficients (chi^2^ = 36.206, p < 0.001), and an overall classification accuracy of 67.1%.

**Table 4:** Factors associated with Tribenuron-methyl (TBM) resistance in wild mustard populations.

Variables	B	SE*	Wald	df	Sig.	Exp(B)
**Number of chickpea cultivation in the last 5 years**						
Two times vs. Three times	−0.653	0.254	6.614	1	0.010	0.520
**Fertilizer**			1.237	2	0.539	
P vs. P&K	0.523	0.637	0.674	1	0.411	1.688
N&P vs. P&K	0.641	0.600	1.140	1	0.286	1.898
**Wheat cultivar**						
Azar-2 vs. Sardari	0.797	0.286	7.752	1	0.005	2.218
**Sowing date**	0.414	0.262	2.494	1	0.114	1.512
November vs. October						
**Application frequency of TBM in the last 5 years**			10.283	3	0.016	
Single application (1×) vs. No application	0.592	0.609	0.944	1	0.331	1.807
Dual applications (2×) vs. No application	0.855	0.646	1.751	1	0.186	2.351
Triennial applications (3×) vs. No application	2.366	0.832	8.083	1	0.004	10.651
Constant	−1.616	0.775	4.350	1	0.037	0.199

* SE= Standard Error.

The logistic regression analysis showed that the frequency of TBM application had the greatest impact on survival development, followed by wheat cultivar selection and crop rotation practices (Table 4). While one time and two times TBM applications did not show statistically significant effects, three times application significantly increased the probability of probability of survival compared with no TBM application. This indicates that wild mustard populations exposed to TBM three times during the last five years were 10.6 times more likely to exhibit field resistance compared to populations with no herbicide application (Table 4). One-time and two-time applications of TBM application during the last 5 years increased the probability of survival occurrence by 2.3 and 1.80 times, respectively, compared to no TBM application. These results show a non-linear, cumulative use intensity relationship between TBM application frequency and survival evolution, with frequent exposure triggering exponential increases in selection pressure. The data underscore the necessity of restricting TBM use to biennial or less frequent intervals within integrated weed management frameworks, thereby mitigating the enrichment of less-sensitive biotypes through prolonged herbicide selection (Table 4).

The choice of wheat cultivar also played a significant role in survival development, though its impact was secondary to herbicide application frequency. Compared to the Sardari cultivar, the use of Azar-2 significantly increased the probability of survival 2.2-fold (Table 4).

Increasing the frequency of chickpea cultivation from two to three times in the last five years significantly reduced the probability of wild mustard resistance to TBM. With three cultivations of chickpeas during the last five years decreasing survival odds by a factor of 0.5. This finding highlights the role of diversified crop rotations in disrupting the life cycle of wild mustard and reducing the selection pressure for reduced sensitivity (Table 4).

The type of fertilizer used (phosphorus or nitrogen and phosphorus compared to potassium and phosphorus) and sowing date (November compared to October) did not show statistically significant effects on the probability of survival. However, the odds ratios for these factors (Exp(B) = 1.7 for phosphorus and 1.898 for nitrogen and phosphorus; Exp(B) = 1.5 for November sowing) suggest potential trends of resistance and sensitivity probability, possibly due to differences in crop and weed competition conditions (Table 4).

### 3.2 The effects of the farmers’ characteristics on the field resistance of wild mustard to TBM

The reduced sensitivity to TBM was significantly affected by the farm size (including 1–5, 5.1 to 10, 10.1 to 15, and above 15 ha), while the other farmer-related characteristics (including experience, level of education, and having a secondary occupation) did not have a significant effect on the survival rate of wild mustard to TBM (Table 5). The highest recorded survival proportion of wild mustard to TBM (42.1%) was observed in farms with an area of more than 15 ha compared to the other sizes of farms (Table 6).

**Table 5:** Results of the Mann-Whitney U Test and Kruskal-Wallis H test for comparing two and multiple independent groups of agronomic practices.

Non-Parametric Tests	Groups	df	Test statistics	P-value
Mann-Whitney U test	Farmer occupation	1	11924.00	0.906
			
			
Kruskal-Wallis H test	Farmer experience	4	2.53	0.639
	Farmer education level	4	6.03	0.197
	Field area	3	10.27	0.016

**Table 6:** Descriptive statistics to compare the median, ranks, and mean of frequency of resistant populations across the fields with varying area.

Field area (ha)	Median (%)	Mean Rank	Resistance proportion ± SE*
1 to 4.9	16.67	145.01	23.1 ± 2.5
5 to 9.9	25.00	157.94	26.0 ± 2.3
10 to 14.9	20.00	155.21	25.9 ± 4.7
15 ha and more	37.00	200.09	42.1 ± 5.8

* SE= Standard Error.

## 4. Discussion

The findings of this survey indicate a concerning and well-established occurrence of wild mustard survival following TBM application throughout the study region. A substantial portion of fields (38.7%) exhibit high survival proportions. While nearly half of the fields still display lower resistance, this mixed distribution pattern suggests that reduced sensitivity is at a critical and evolving stage, driven by ongoing selection pressure from repeated herbicide applications. This highlights the urgent need to discuss the agronomic factors contributing to this trend and to explore management strategies to mitigate the further spread of resistance.

The evolution of TBM reduced sensitivity in wild mustard in the studied dryland wheat fields is closely linked to specific agronomic practices. We have clarified the relative contributions of herbicide use frequency, wheat cultivar selection, and crop rotation to the wild mustard reduced sensitivity. These findings confirm regional trends and offer practical insights for landscape-scale management.

The evolution of TBM reduced sensitivity in wild mustard in the studied dryland wheat fields is closely linked to specific agronomic practices, explicitly highlighting the relative contributions of herbicide use frequency, wheat cultivar selection, and crop rotation. Our results highlight that the frequency of TBM application is the most significant factor associated with increased survival proportion in wild mustard populations. Fields with a history of repeated TBM use exhibited the highest survival rates, supporting the well-established principle that recurrent use of herbicides with the same mode of action exerts strong selective pressure on weed populations [30,31].

This is consistent with findings from other regions, where resistance to ALS-inhibitor herbicides has been shown to develop rapidly, sometimes within as few as four to five applications. The rapid evolution of resistance in response to repeated ALS-inhibitor use has been well documented. For instance, continuous and intensive reliance on TBM rapidly depletes susceptible individuals and imposes strong directional selection pressure, culminating in the fixation of highly resistant *Sinapis alba* biotypes within only five consecutive years (Rosario et al., 2011; Cruz-Hipolito et al., 2013). ALS-inhibitor resistance in Kochia (*Kochia scoparia* L. Schrad.) was first observed just five years after these herbicides were introduced [32]. Similarly, Beckie and Reboud [33] reported that resistance in field pennycress (*Thlaspi arvense* L.) increased dramatically after four applications of ALS inhibitor herbicide (ethametsulfuron). They found that the resistance level in the seed bank increased from 29% of recruited seedlings after one application to 85% after four applications of the ALS inhibitor. Furthermore, Cruz-Hipolito et al. [34] demonstrated that wild mustard populations subjected to annual TBM applications over five consecutive years evolved pronounced target-site resistance driven by an amino acid substitution (Pro197Ser) within the ALS enzyme, yielding a resistance factor of 9.1. Moreover, Rosario et al. [35] confirmed that resistant wild mustard biotypes selected under continuous tribenuron-methyl pressure not only exhibit extreme target-site resistance, but also demonstrate broad cross-resistance to multiple chemical families of ALS inhibitors, such as other sulfonylureas, triazolopyrimidines, and imidazolinones. Building upon these evolutionary dynamics, recent investigations reveal that *S. alba* resistance frequently involves complex multi-factorial adaptations rather than simple point mutations. For instance, while Palma-Bautista et al. [36] demonstrated that resistant biotypes sustain high-level survival against TBM with minimal variation in absorption or translocation, Chtourou et al. [37] established that populations can evolve extreme resistance factors exceeding 144-fold. Through molecular modeling, they proved that key amino acid substitutions (such as Pro197Ser and Trp574Leu) significantly disrupt hydrogen bonding and impair binding affinity within the ALS active site for both sulfonylureas and imidazolinones. Moreover, the partial reversal of resistance via cytochrome P450 inhibitors underscores the critical coexistence of non-target-site mechanisms (NTSR) alongside TSR. Consequently, this convergence of multi-factorial adaptations highlights a severe agronomic risk, shifting the evolutionary trajectory toward multi-family resistance frameworks that necessitate robust integrated and rotational weed management strategies.

Herbicide rotation (alternating products with different sites of action across seasons) has been shown to reduce selection pressure and delay resistance evolution [30,38]. Thus, the utilization of herbicide rotations as a tactic to delay such resistance is one of the best strategies [33]. Applying herbicides with different modes of action in herbicide rotation reduces selection pressure, thereby restricting the evolution of herbicide resistance [31]. Therefore, to avoid herbicide resistance evolution in the near future, farmers should follow herbicide rotation as an effective strategy [39]. in the study of Hada et al. [38], less occurrence of *G. coronaria* resistance to ALS-inhibiting herbicides was associated with more herbicide rotation. Our data, however, indicate that herbicide rotation remains underutilized in the study region, as also reported in previous Iranian studies [12,40].

Beyond chemical control, our study demonstrates that wheat cultivar selection significantly influences the survival proportion to TBM. Fields planted with the Sardari cultivar exhibited lower survival proportion compared to those with Azar-2. This may indicate possible differences in crop competitiveness between cultivars-attributes documented in previous research [41,42] which likely contribute to its effectiveness in suppressing wild mustard and reducing the weed seed bank. This finding reinforces the value of crop competitiveness, which has become a focal point in resistance management research prompted by the growing complexity of herbicide resistance in weed populations [18,43]. Competitive cultivars can suppress weed emergence and growth, reducing both the need for herbicide applications and the likelihood of resistance evolution [44,45]. Indeed, traits such as early vigor, high tillering capacity, and increased plant height have all been linked to improved weed suppression [19,44]. Consequently, the development of weed-suppressive crop genotypes is an essential strategy for herbicide-resistant weed management, as enhancing crop competitiveness reduces herbicide selection pressure, weed seed production, and the soil seed bank [46].

Crop rotation emerged as another key factor influencing survival dynamics in our study. The inclusion of chickpea in rotation with wheat (practiced in many of the surveyed farms) was associated with lower levels of survival proportion. Chickpea’s spring sowing disrupts the life cycle of wild mustard, providing a window for non-chemical control measures and reducing the selection pressure imposed by continuous wheat cultivation and herbicide use. Numerous investigations have demonstrated that crop rotation serves as a vital strategy for reducing the emergence of herbicide-resistant weed populations [30,43,47]. The integration of diverse crops within rotation systems enhances the efficacy of weed management by leveraging the unique growth patterns and nutrient requirements of different species, ultimately contributing to more sustainable agricultural practices. For example, incorporating spring crops into a rotation with winter crops yields optimal results in managing herbicide-resistant populations of blackgrass (*Alopecurus myosuroides* Huds.) [48], and rotational strategies have been shown to delay or minimize the development of resistance in wild oat (*Avena fatua* L.) [49].

Farm size is considered to be a measure of economic status. Farmers with greater financial resources may invest more intensively in crop management [50], often pursuing higher yields and economic returns through increased input use. Consequently, the frequency of herbicide application tends to rise; our data showed that the relative use of TBM 2 or 3 times over the past five years increased progressively across farm size categories, ranging from 26% in smaller farms to 42.4% in farms exceeding 15 ha (Table 7). This aligns with Herzog et al. [51], who noted a positive correlation between agricultural farm size and management intensity.

**Table 7:** Chi-square distribution table of field area in relation to the use of Tribenuron-methyl in the last 5 years.

	The use of Tribenuron-methyl in the last 5 years							
Field area	No use		One time		Two times		Three times	
	*N*	*%*	*N*	*%*	*N*	*%*	*N*	*%*
1 to 4.9 ha	9	7.3	82	66.7	30	24.4	2	1.6
5 to 9.9 ha	7	5.7	79	64.8	31	25.4	5	4.1
10 to 14.9 ha	1	2.9	21	60	9	25.7	4	11.4
15 ha or more	1	3	18	54.5	6	18.2	8	24.2
**P-value**	0.001							

A comparison of fields revealed a temporal shift in herbicide choice: in the year preceding the experiment, 49.1% of wheat fields received TBM, while 45.2% received applications of 2,4-D and 5.7% of fields were sprayed with a combination of both herbicides. During the experimental year, reliance shifted predominantly to 2,4-D (67.7%) and tank mixtures, with only 2.3% applying TBM alone. This transition reflects growing local awareness of declining TBM efficacy. However, despite this adaptive shift, farmers in the study region still predominantly depend on chemical control [8]. Given the variability in growers’ resistance perception, strengthening targeted extension programs is crucial to facilitate proactive management before resistance becomes widespread.

Methodologically, while plant material was authenticated using standardized taxonomic keys, morphological uniformity does not preclude underlying genetic divergence. The potential presence of micro-morphological variants may partially account for the observed spectrum of survival proportions. Furthermore, regional studies have documented emerging TBM resistance driven by localized selection pressures [10,52]. The potential co-existence of target-site mutations and non-target-site mechanisms, as highlighted by Palma-Bautista et al. [36], Chtourou et al. [37], underscores the need for future molecular profiling to precisely map population structures and resistance evolution.

Given the continued heavy reliance on chemical weed control in Iranian wheat systems [8], bridging the gap between scientific recommendations and on-farm practices is paramount. Strengthening targeted extension programs to enhance growers’ awareness of diversified weed management will be pivotal in mitigating the future evolution and spread of herbicide resistance.

## 5. Conclusion

This large-scale study demonstrates that the evolution of TBM reduced-sensitivity in wild mustard across dryland wheat systems is strongly driven by intensive herbicide application and specific agronomic choices such as crop rotation and cultivar selection. Our findings highlight the complex interplay of factors driving resistance and underscore the necessity of diversified management strategies to minimize herbicide selection pressure. Consequently, mitigating resistance requires shifting toward Integrated Weed Management (IWM). Given the scarcity of alternative herbicides, overcoming regional challenges depends heavily on strengthening extension programs, enhancing grower awareness through targeted training, and fostering collaborative knowledge exchange. Ultimately, empowering farmers with practical, science-based agronomic frameworks is essential to preserve herbicide efficacy, protect livelihoods, and secure long-term wheat productivity.

## Supporting information

S1 Data Raw experimental data. (XLSX)
